# WHAT AND HOW DID IT WORK? A SYSTEMATIC REVIEW OF THE INTERVENTION STUDIES OF CHANGING PERCEPTION TOWARD OLDER ADULTS

**DOI:** 10.1093/geroni/igac059.1270

**Published:** 2022-12-20

**Authors:** Yeonjung Lee, Lun Li, Laxman Shrestha

**Affiliations:** Chung-Ang University, Seoul, Seoul-t'ukpyolsi, Republic of Korea; MacEwan University, Edmonton, Alberta, Canada; University of Calgary, Calgary, Alberta, Canada

## Abstract

Social workers are one of the major professionals serving for older adults. However, social work students often rank gerontology at the bottom of their future professional practice. Studies show that students have negative attitudes about, and perceptions of older adults and aging. Such stereotypes result in social work students considering practice areas other than gerontological social work. The objective of this study is to conduct a systematic review and identify any types of the intervention studies of changing perception and attitudes toward older adults and aging among social work students. This systematic review identified empirical research studies written in English that were published in a peer-reviewed journal before November 2021. Systematic search was carried out within four electronic databases (AgeLine, Education Research Complete, Social Work Abstracts, SocINDEX with full text) with the key words such as “social work student” and “aging or ageing or elderly or older adults or seniors or geriatrics or gerontology”. A total of 470 abstracts were identified, and after careful review of 143 full articles yielded from the databases, 43 journal articles were included in this study. Analysis of the selected literature provides that major interventions have been tried include service learning, lecture, curriculum development, simulation, training program, and intergenerational program. This systematic review highlights the importance of the experiential learning, partnership development with community, and interprofessional collaboration to provide the best model of practice for changing perception toward aging and older adults among social work students.

